# Perception of Orofacial Esthetics Using a Scan‐Free Virtual Video Mock‐Up: A Comparative Analysis of Self‐Evaluation Versus Unknown‐People Assessment

**DOI:** 10.1111/jerd.70059

**Published:** 2025-11-26

**Authors:** Aspasia Pachiou, Miha Pirc, Tim Joda, Ronald E. Jung, Alexis Ioannidis

**Affiliations:** ^1^ Clinic of Reconstructive Dentistry Center for Dental Medicine, University of Zurich Zurich Switzerland; ^2^ Department of Reconstructive Dentistry University Center for Dental Medicine, University of Basel Basel Switzerland

**Keywords:** digital dentistry, digital diagnostics, digital smile design, orofacial esthetic scale, virtual mock up

## Abstract

**Objective:**

To evaluate perceptions of scan‐free virtual video mock‐ups using the Orofacial Esthetic Scale (OES), comparing self‐assessments and evaluations of anonymized individuals by dental professionals and laypeople.

**Materials and Methods:**

This study was conducted at the Clinic of Reconstructive Dentistry, University of Zurich. Fifty participants (25 dental professionals and 25 laypeople) were video recorded under standardized conditions while smiling, turning from en face to profile, and speaking to simulate dynamic facial movements. Recordings were obtained using a clinical capture application linked to the device's native rear camera, and AI‐generated scan‐free virtual video mock‐ups were returned within minutes. Each participant rated their own mock‐up using the Orofacial Esthetic Scale (OES) and, in randomized order, evaluated five anonymized virtual mock‐ups generated from the software library (based on real patient recordings but fully de‐identified for privacy protection). Median OES scores were compared using Wilcoxon signed‐rank and Mann–Whitney *U* tests (*α* = 0.05).

**Results:**

Fifty participants (25 dentists and 25 laypeople) were included. Overall, ratings improved from baseline to the scan‐free virtual video mock‐up for tooth color and alignment (*p* = 0.003 and *p* = 0.014), while no significant changes were observed for the remaining items. In subgroup analyses, laypeople reported significant improvements in tooth color, alignment, form, and overall esthetics after Holm correction, whereas no differences were found among dentists. For mock‐up ratings, laypeople assigned higher scores than dentists across all OES items (all *p* < 0.05), except for tooth alignment and facial profile. When assessing anonymized mock‐ups of other individuals, laypeople consistently rated all eight items significantly higher than dentists.

**Conclusions:**

Within the limitations of this study, it was concluded that lay participants assigned higher esthetic ratings than dental professionals for virtual video mock‐ups of themselves and of unknown individuals. Comparisons between self‐evaluation and evaluation of anonymized individuals showed no consistent differences among dentists, while several item‐level differences were observed among lay participants. Compared with natural dentition, ratings for tooth color and tooth alignment increased after viewing the virtual mock‐up, while other items did not change significantly.

**Clinical Significance:**

These findings suggest that integrating simplified digital simulations with patient‐reported outcome measures enhances communication, aligns expectations, and supports patient‐centered shared decision‐making in prosthodontic and implant treatment planning.

## 1 Introduction

The assessment of orofacial esthetics is a cornerstone in prosthodontics, playing a pivotal role in shaping patient satisfaction and influencing clinical outcomes [1]. Esthetics are not merely superficial concerns but integral components of psychosocial well‐being, as facial and dental appearance significantly affect self‐esteem, social interactions, and quality of life [2]. Recent advances in digital technologies have expanded the possibilities for esthetic evaluation through virtual designs and video‐based simulations [3]. Previous studies assessing orofacial esthetics have relied primarily on static photographic evaluations, including comparisons across different dental specialties and analyses of esthetic characteristics of the teeth and surrounding anatomical structures [4, 5].

Virtual mock‐ups allow for the visualization of proposed esthetic outcomes, enabling patients to preview changes before treatment initiation [4, 5]. Digital Smile Design (DSD) and related mock‐up platforms facilitate visualization and clinician–patient communication and have been associated with higher patient engagement and treatment acceptance [6, 7, 8]. During diagnosis and planning, dynamic previews function as adjuncts, particularly in scan‐light or scan‐free workflows, and should be interpreted as representational rather than definitive [9, 10]. This innovation represents a shift from conventional static models to dynamic, customizable representations of potential results [11]. It also facilitates the concept of minimally invasive approaches and individualized treatment planning [12]. Despite these advancements, there is limited understanding of how different populations, including dental professionals and laypeople, perceive these digital designs. Research indicates that factors such as professional expertise, gender, age, and prior treatment experience influence esthetic perceptions, leading to variations in evaluations between clinicians and the general public [13, 14]. These differences highlight the need for a deeper exploration of esthetic preferences across diverse groups to better integrate digital esthetic technologies into clinical practice [15].

Understanding the interplay between individual perceptions and societal norms is essential for enhancing communication and shared decision‐making in prosthodontics [9, 10]. A thorough comparison of esthetic assessments between experts and laypeople reveals potential gaps in perception, guiding improvements in treatment planning and patient education [16, 17]. Professional training often results in clinicians adopting narrower thresholds of esthetic acceptability, leading to more critical evaluations of smile features compared with lay observers [18, 19, 20, 21, 22, 23, 24, 25]. Although professionals apply stricter thresholds, their ratings typically follow consistent internal criteria rather than reflecting a blanket bias toward or against specific visualization methods [26]. Additionally, exploring the relationship between self‐evaluations and external assessments will provide insight into patients' self‐awareness and expectations regarding their orofacial appearance.

The Orofacial Esthetic Scale (OES) has emerged as a standardized and validated tool to evaluate patients' perceptions of their dental and facial appearance. This scale was developed to address the subjective nature of esthetic evaluations and provide an objective framework for assessment [27]. The OES demonstrates high reliability and validity across diverse populations, making it an invaluable metric for both research and clinical practice [28, 29]. Studies have shown that the OES is sensitive to both objective dental changes and subjective perceptions, ensuring its utility in evaluating the impact of esthetic interventions [30]. Its adoption has enhanced the ability of clinicians to align treatment objectives with patients' esthetic expectations, thereby improving patient‐centered care and fostering better communication between patients and providers [31, 32, 33].

Emerging technologies are exploring the possibility of creating visualizations based solely on video recordings, which significantly reduce costs [16]. This approach eliminates the need for time‐consuming intraoral scans and allows for real‐time presentation of treatment options without additional expenses [3, 15].

Therefore, it was the aim of this study to evaluate perceptions of scan‐free virtual video mock‐ups using the Orofacial Esthetic Scale (OES), comparing self‐assessments and evaluations of anonymized individuals by dental professionals and laypeople. Based on previous reports that dental professionals are generally stricter than laypeople when evaluating smile esthetics, and that virtual visualizations may improve esthetic perception, it was hypothesized that: (1) lay participants would assign higher esthetic ratings than dental professionals for virtual video mock‐ups, (2) differences would exist between self‐evaluation and the evaluation of unfamiliar individuals, and (3) virtual video mock‐ups would increase esthetic ratings compared with natural dentition.

## 2 Materials and Methods

This study was conducted at the Clinic of Reconstructive Dentistry, University of Zurich under standardized conditions between May and August 2025.

This study was reviewed by the Ethics Committee of Kanton Zurich, which determined that it does not fall under the Swiss Human Research Act (HRA); therefore, no formal ethics approval was required. Data handling complied with institutional policy and the Swiss Federal Act on Data Protection (FADP); all analyses used anonymized records. Participants were anonymized prior to analysis.

### 2.1 Participants

Fifty adults were enrolled: 25 dental professionals (dentists) and 25 laypeople. Eligibility criteria included age ≥ 18 years and ability to provide consent. Exclusion criteria were facial deformity affecting esthetic assessment, ongoing extensive dental treatment, or unwillingness to be video‐recorded. Group allocation (professional vs. laypeople) was based on occupation/self‐report. Participants were randomized 1:1 to assessment order: Order‐A (self mock‐up—unknown people) or Order‐B (unknown people—self mock‐up).

### 2.2 Video Acquisition (Dynamic Smiles)

Each participant was video‐recorded using the latest model of a standard tablet (iPadOS, Apple, Cupertino, CA, USA) and the Invisalign Practice App, a dedicated clinical capture application (Align Technology, Tempe, AZ, USA), which accessed the device's native rear camera. No in‐app processing or filters were enabled. Following a standardized script, participants smiled broadly, slowly turned from en face to profile views, and spoke briefly to simulate dynamic facial movements. All recordings were performed by a single examiner trained and calibrated on the protocol of the clinic, ensuring consistent instructions, framing, and timing across participants. Sessions were completed with fixed lighting, a neutral background, and consistent camera‐to‐subject distance/framing per protocol.

Videos were submitted via the application, which returned AI‐generated scan‐free virtual video mock‐ups within minutes. Videos were submitted via the Invisalign Practice App, which generated AI‐created, scan‐free virtual video mock‐ups within minutes. According to the manufacturer, the ortho‐restorative simulation is AI‐driven and leverages a large case library (> 19 million prior Invisalign treatments) to propose tooth alignment and color/shape adjustments and to suggest combined orthodontic–restorative treatment options. In this study, the software output was used strictly as an esthetic communication mock‐up; no treatment was planned or initiated based on the simulation.

For between‐person assessments, participants evaluated a fixed set of five anonymized mock‐ups of unfamiliar individuals sourced from the software's de‐identified case library. These mock‐ups originate from real patient recordings but undergo automated facial de‐identification (e.g., modification of unique facial features and skin details) to ensure non‐traceability and GDPR‐compliant secondary use. The set was pre‐selected to avoid duplicates and artifacts and was displayed without demographic information. The order of the five mock‐ups and the sequence of self versus unknown people's assessments were computer‐randomized.

### 2.3 Outcome Measures

To capture both baseline and mock‐up perceptions, participants completed the Orofacial Esthetic Scale (OES) in the following sequence:

1. Baseline self‐assessment: participants rated their own natural dentition using the OES.
2. Self mock‐up assessment: participants rated the AI‐generated scan‐free virtual video mock‐up of themselves using the OES.
3. Assessment of unknown people: participants rated five anonymized mock‐ups of unfamiliar individuals using the OES (order randomized).

The OES comprises eight items scored on an 11‐point scale (0–10); higher scores indicate better orofacial esthetics. The primary comparison contrasted professionals versus laypeople for selected esthetic aspects (tooth form, gingival appearance, and overall esthetics). Secondary comparisons included within‐subject differences between natural dentition versus self mock‐up and self versus unknown people's assessments.

### 2.4 Sample Size Calculation

Because no prior studies have evaluated orofacial esthetics using an AI‐generated, scan‐free virtual video mock‐up, effect sizes were unavailable. Therefore, a pragmatic total sample of 50 participants (25 dentists, 25 laypeople) was adopted to (i) ensure balanced groups for between‐cohort contrasts, (ii) obtain stable variance and feasibility estimates to inform a definitive study, and (iii) maintain reasonable sensitivity to medium standardized effects while minimizing resource waste [34, 35]. This sample also meets common recommendations for pilot/novel methods studies assessing perceptual outcomes, balancing feasibility with precision [34]. Equal allocation also limits small‐sample bias in nonparametric rank tests and simplifies interpretation.

### 2.5 Statistical Analysis

Data were screened for range violations (OES 0–10), missingness, and outliers (box‐plot/IQR rule). Normality of item‐level and composite OES scores was assessed using Shapiro–Wilk tests with visual inspection of histograms. Given the ordinal scale of the OES and frequent departures from normality, nonparametric tests were prespecified. Median (IQR) scores are reported. Within‐subject contrasts (natural dentition vs. self mock‐up; self mock‐up vs. unknown people) used Wilcoxon signed‐rank tests; between‐group contrasts (professionals vs. laypeople) used Mann–Whitney *U* tests. For within‐subject subgroup analyses across the eight OES items, multiplicity was controlled using the Holm method (*α* = 0.05). Two‐tailed *α* = 0.05. Where appropriate, rank‐biserial effect sizes (*r*) with 95% CIs are provided. Analyses were performed in R (version 2025.05.1 + 513; R Foundation for Statistical Computing, Vienna, Austria).

Gender‐based analysis was not included, as the study was neither powered nor designed to detect gender effects, and subgroup testing risks spurious interpretation outside the scope of the research question.

## 3 Results

Fifty adults completed the study and were analyzed (25 dentists, 25 laypeople). All recordings were obtained by a single examiner under standardized conditions; no sessions were excluded for protocol deviations. Participant demographics included 64% women and 36% men. No missing data were imputed. Assessment‐order randomization was implemented as planned.

### 3.1 Baseline OES (Natural Dentition)

At baseline, central tendencies on the 0–10 OES clustered high (medians generally 7–8), with the highest central tendency for gum appearance (median 8 [Q1: 7.25, Q3: 9]). Representative values included tooth color median 7 (Q1: 6, Q3: 8), tooth alignment median 7 (Q1: 6, Q3: 9), and overall esthetics median 7 (Q1: 7, Q3: 8).

### 3.2 Self‐Assessment Compared With Evaluation of Anonymized Individuals

When comparing self‐assessments with evaluations of anonymized mock‐ups, participants rated their own virtual mock‐ups at medians of 8 for all OES items, whereas anonymized individuals were also rated at medians of 8 for all OES items (Wilcoxon signed rank test—all *p* > 0.05 except mouth appearance—*p* = 0.047).

### 3.3 Within‐Subject Change: All Participants

**FIGURE 1:**
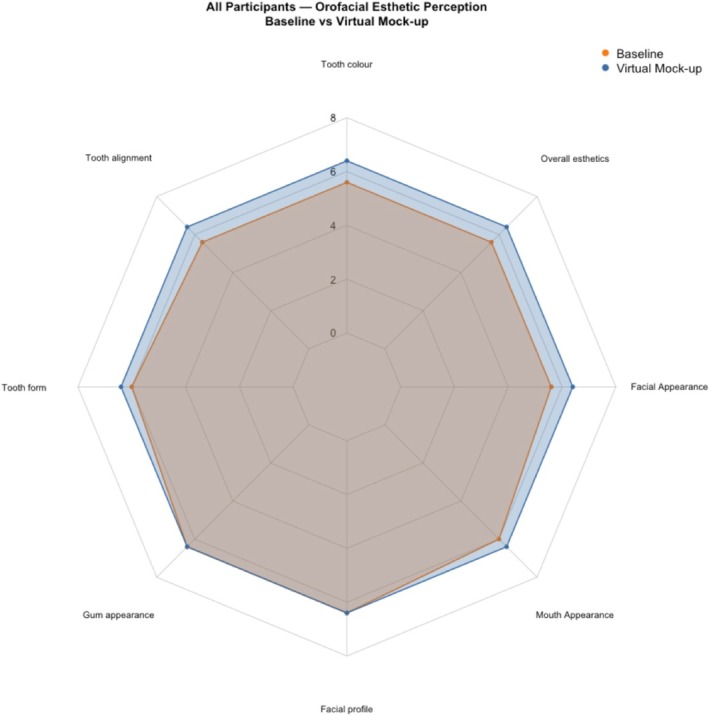
OES assessments of the natural dentition compared to the virtual mock‐up, tooth color and tooth alignment increased significantly (Wilcoxon signed‐rank: *p* = 0.003 and *p* = 0.014).

When comparing the OES assessments of the natural dentition to the virtual mock‐up, tooth color and tooth alignment increased significantly (Wilcoxon signed‐rank: *p* = 0.003 and *p* = 0.014, respectively); for all other items there was no statistically significant difference detected (all *p* > 0.05) (Figure 1).

### 3.4 Within‐Subject Change by Group

**FIGURE 2:**
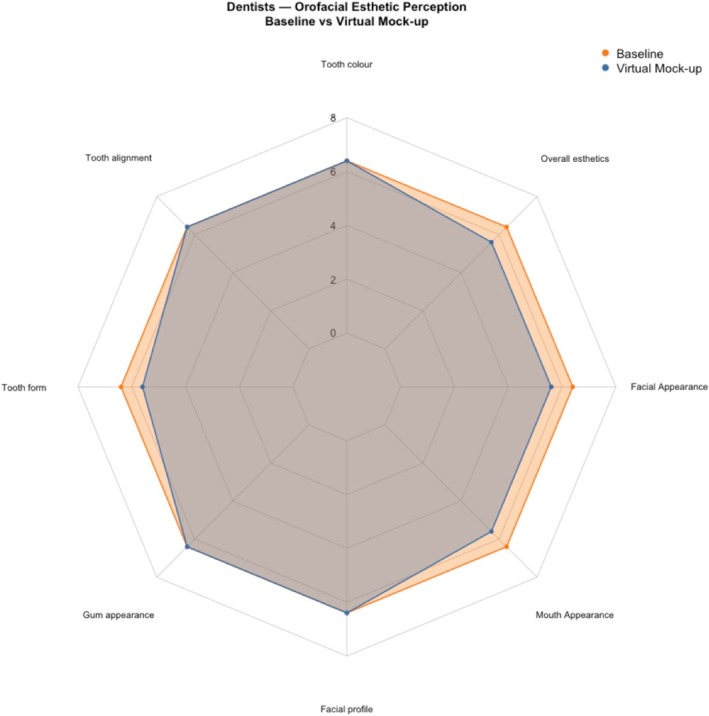
Comparing the assessment of natural dentition and virtual mock‐ups among dentists.

Among dentists, no item remained significantly different after multiplicity control across eight items when comparing the assessment of natural dentition and virtual mock‐ups (Holm; all adjusted *p* ≥ 0.35). The only unadjusted signal was a decrease for mouth appearance (Hodges–Lehmann [HL] Δ = −1.00; 95% CI, −1.50 to −0.00; *p* = 0.044; Holm‐adjusted *p* = 0.35). For all other items, estimates were small with 95% CIs crossing 0 (Figure 2).

**FIGURE 3:**
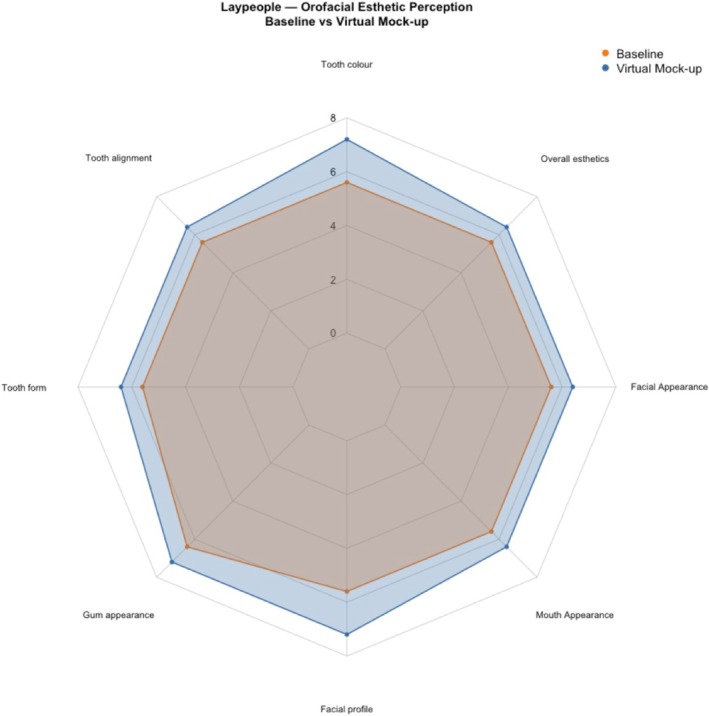
Comparing the assessment of natural dentition and virtual mock‐ups among laypeople.

In contrast, laypeople showed statistically significant improvements in their assessments of natural dentition and virtual mock‐ups after Holm correction for tooth color (OES 1) (HL Δ = +2.5; 95% CI, +2.0 to +3.0; *p* < 0.001; adjusted *p* = 0.002), tooth alignment (OES 2) (Δ = +2.0; 95% CI, +1.0 to +3.0; *p* = 0.002; adjusted *p* = 0.02), tooth form (OES 3) (Δ = +2.0; 95% CI, +1.0 to +3.0; *p* = 0.01; adjusted *p* = 0.048), and overall esthetics (OES 8) (Δ = +1.5; 95% CI, +0.5 to +2.5; *p* = 0.01; adjusted *p* = 0.04). Gum appearance (OES 4), facial profile (OES 5), mouth appearance (OES 6) and facial appearance (OES 7) favored improvement numerically (HL = +1.0 to +1.5) but were not significant after correction (all adjusted *p* > 0.05) (Figure 3).

**FIGURE 4:**
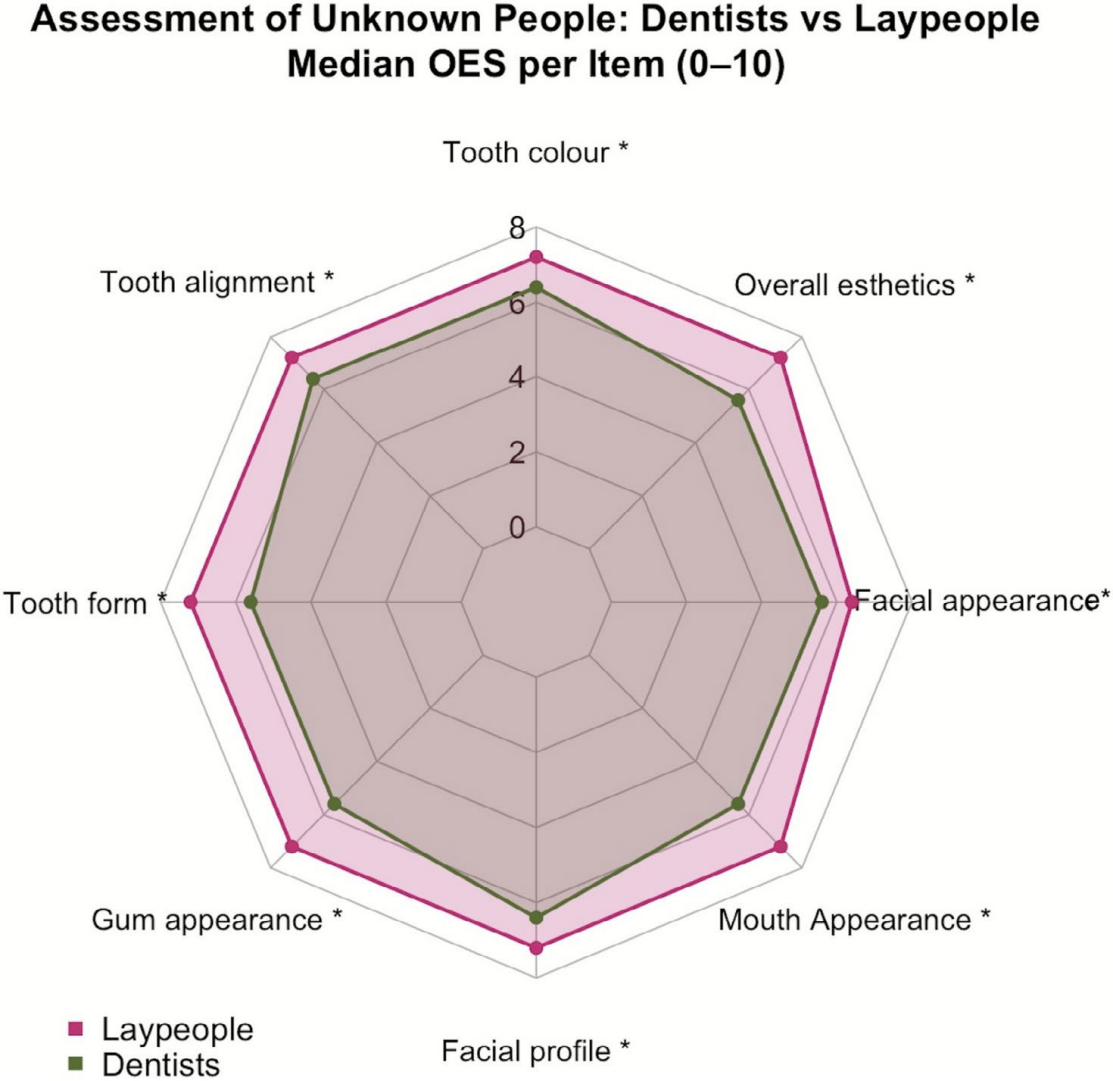
Laypeople consistently assessed higher unknown people's smiles than dentists (*p* < 0.05). Statistical significance is indicated with *.

**FIGURE 5:**
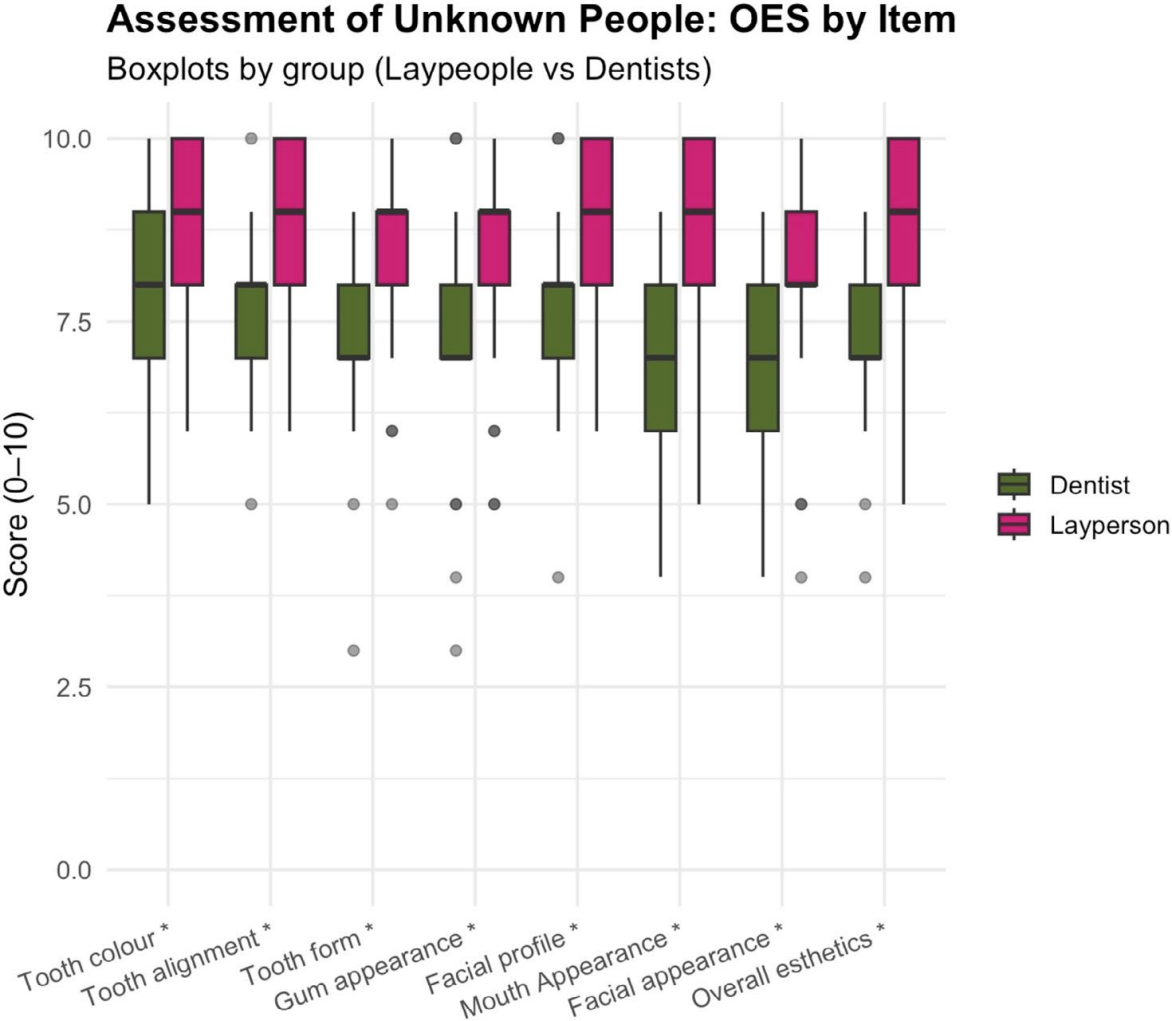
Laypeople consistently assessed higher unknown people's smiles than dentists (*p* < 0.05). Statistical significance is indicated with *.

Between‐group analyses indicated that laypeople scored statistically significantly higher than dentists in evaluations of anonymized unknown individuals (Wilcoxon signed rank with continuity correction—all *p* < 0.05; *p* value range < 0.001) (Figures 4 and 5).

### 3.5 Between‐Group Comparisons on Virtual Mock‐Ups

**FIGURE 6:**
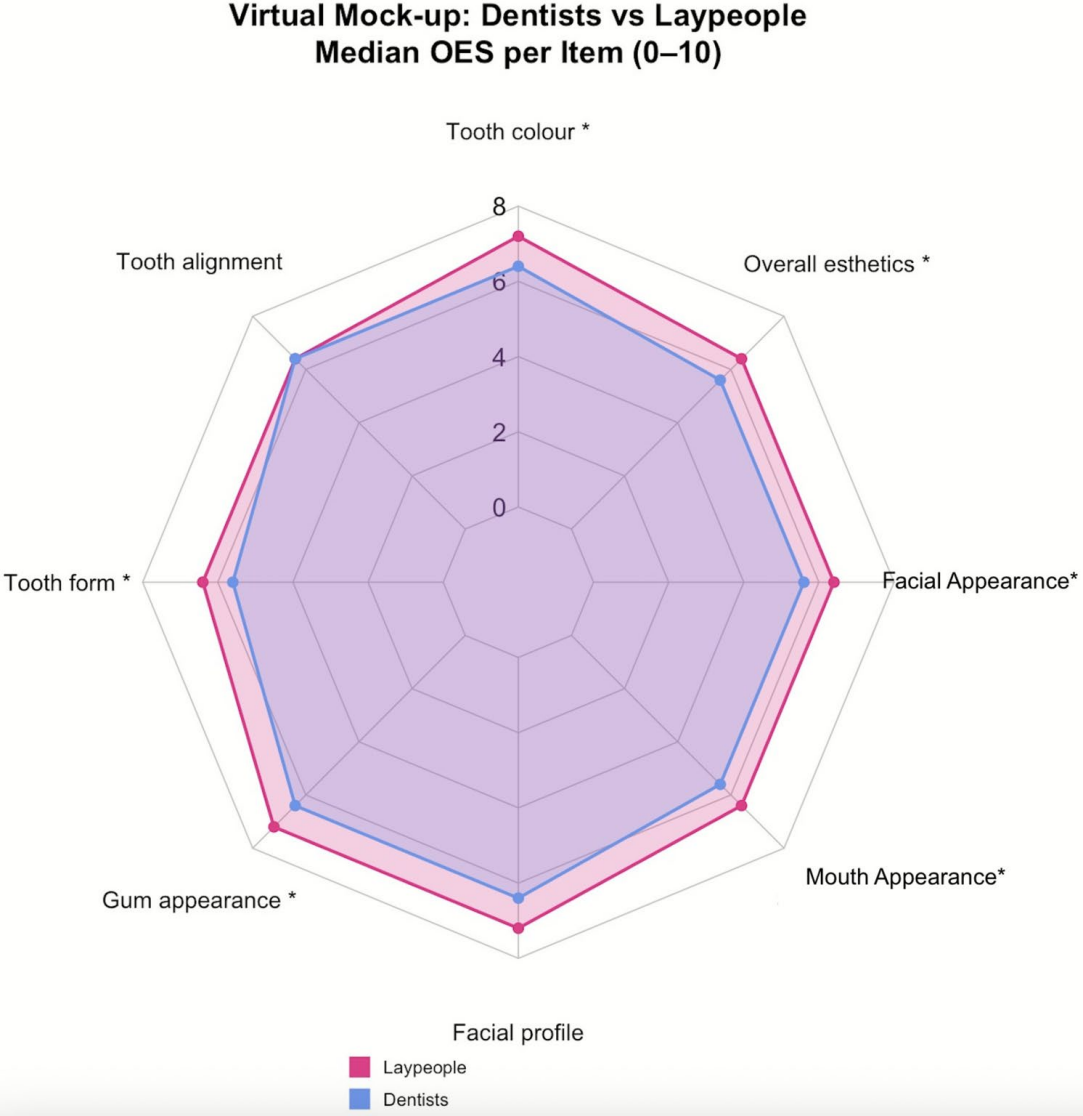
Comparing the assessment of virtual mock‐ups among laypeople and dentists. Laypeople consistently rated higher than dentists. Statistical significance is indicated with *.

**FIGURE 7:**
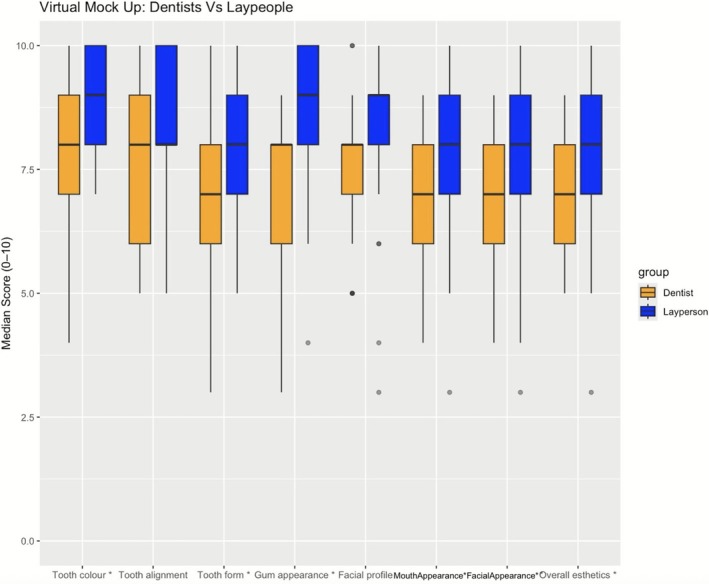
Comparing the assessment of virtual mock‐ups among laypeople and dentists. Laypeople consistently rated higher than dentists. Statistical significance is indicated with *.

For the virtual mock‐up ratings, laypeople scored higher than dentists on OES‐1, OES‐3, OES‐4, OES‐6, OES‐7, and OES‐8 (Mann–Whitney *U*: *p* = 0.025, 0.005, 0.006, 0.047, 0.037, and 0.006, respectively). Assessments of OES‐2 and OES‐5 did not differ between groups (*p* = 0.067 and 0.053) (Figures 6 and 7). These between‐group analyses were exploratory and are presented unadjusted.

#### 3.5.1 Clean Baseline Results

*Multiplicity and reporting*. Within‐subject subgroup analyses controlled itemwise multiplicity with Holm (*α* = 0.05); all tests were two‐sided, with HL median change and 95% CIs reported where applicable.

## 4 Discussion

This comparative study predominantly revealed: (i) high OES scores for natural dentition at baseline; (ii) self‐assessments compared with evaluations of anonymized individuals showed [to be completed once values are inserted]; (iii) significant improvements for tooth color and tooth alignment when comparing natural dentition with video mock‐ups; and (iv) improvements across multiple OES items among laypeople.

The comparison between self‐assessments and evaluations of anonymized individuals showed that participants were equally critical when assessing themselves. This finding suggests that the perception of one's own esthetics differs from the evaluation of unknown people, with potential influence from self‐awareness, personal bias, or social comparison. Such differences underline the importance of considering both self‐perception and external assessment when interpreting patient‐reported outcomes in esthetic dentistry.

When analyzing the full cohort, the mock‐up modestly improved ratings for the tooth color and tooth alignment (OES‐1, OES‐2). Stratified analyses showed that laypeople perceived broader benefits (OES‐1, ‐2, ‐3, ‐8 after multiplicity control) of the virtual mock‐up, whereas dentists showed no significant item‐level improvements when comparing their natural dentition with their virtual simulation. Between groups, laypeople assigned higher mock‐up ratings than dentists for most OES items, and this pattern extended to evaluations of anonymized “unknown” people. These findings suggest that video‐based digital previews align more closely with lay preferences than with professional benchmarks, underscoring the importance of explicit discussion about esthetic goals during shared decision‐making.

The direction and magnitude of these differences are consistent with prior work showing that clinicians are typically more critical than laypersons when judging smile features and acceptable ranges of variation [16, 18]. Classic and contemporary studies report lower tolerance among professionals for discrepancies in anterior tooth proportions, gingival display, smile arc, and buccal corridors, whereas lay thresholds are generally broader [19, 20, 21, 22, 23]. Recent syntheses also indicate that group background (professional vs. lay), demographics, and cultural context modulate esthetic judgments, which helps explain why in the present laypeople cohort rated both self and unfamiliar mock‐ups more favorably [24, 25].

Notably, even though dentists were stricter raters overall, their evaluations of the mock‐ups were closely aligned with how they judged their own baseline appearance, suggesting internal consistency rather than a categorical bias against the digital preview [26]. This pattern implies that the method efficiently surfaces preference gaps (clinician vs. patient) while remaining acceptable to professionals, thereby supporting expectation alignment, and informed consent in a fast and pragmatic manner.

Methodologically, the OES provided a brief, validated patient‐reported measure tailored to orofacial appearance and supported across populations and languages, enhancing interpretability of item‐level differences, such as for tooth form, gingival appearance, and overall esthetics [27, 28, 29, 30]. Evidence on response formats and short forms further supports pragmatic use in clinical research and quality improvement [29]. The finding that lay ratings improved on overall esthetics (OES‐8) aligns with the construct validity of the OES as a global appraisal metric and with the broader role of PROMs in esthetic and implant dentistry [31, 32].

The study also speaks to emerging digital workflows. Dynamic, video‐based documentation captures lip‐tooth relationships and smile motion better than static images, potentially increasing patient comprehension of proposed changes [9]. Digital Smile Design (DSD) and related mock‐up tools have been shown to aid visualization and communication, often improving engagement and case acceptance; our data suggest that such visualizations particularly resonate with lay viewers [8]. Early evidence assessing users' preferences within DSD environments likewise reports meaningful, but not identical priorities between dentists and laypeople, reinforcing the need for expectation alignment when translating previews into definitive treatment plans [14].

### 4.1 Clinical Implications

Clinicians should anticipate that patients rate video‐based mock‐ups more favorably than providers do, especially for global appearance and tooth form, and proactively calibrate expectations. Structured chairside conversations using PROMs, such as the OES, focus attention on items with the largest expert–lay gaps and document patient priorities before irreversible steps [33]. Furthermore, dynamic previews are a useful adjunct in esthetic counseling, particularly at the diagnostic and planning stages where scan‐light or “scan‐free” approaches are feasible, while recognizing these previews are representational rather than definitive [9, 10]. Turnaround within minutes enabled immediate, low‐burden visualization and use of a validated PROM (OES) in the same session, an approach that can streamline shared decision making by quickly anchoring conversations to patient‐reported priorities and concrete visual scenarios rather than abstract descriptions.

### 4.2 Strengths and Limitations

Strengths include a standardized acquisition protocol, randomization of assessment order, and prespecified nonparametric analyses with multiplicity control. Limitations include the single‐center design, modest sample typical of exploratory work, and implementation of a proprietary AI approach, for which independent, esthetic‐accuracy validation remains preliminary; these factors constrain generalizability. In addition, the assessments were video‐based and did not incorporate intraoral/dental scans, so the workflow cannot be transferred 1:1 to real‐world treatment planning, where comprehensive dentofacial records and functional constraints are required. The study assessed immediate perceptions rather than longitudinal satisfaction or treatment acceptance, and itemwise testing raises the possibility of residual type I error in exploratory between‐group analyses.

### 4.3 Future Directions

Multicenter trials with larger, demographically diverse samples need to examine whether video‐based, scan‐free virtual simulations improve informed consent quality, treatment adherence, and long‐term PROMs. Comparative studies pitting static photographs versus video‐derived frames versus full dynamic clips could quantify modality effects on esthetic judgments [9]. Method comparison against traditional wax‐ups or scan‐based digital mock‐ups helps separate the contribution of motion from that of design. Embedding OES alongside other PROMs and decision‐quality metrics clarifies how best to integrate patient perception into esthetic planning [31].

## 5 Conclusions

Within the limitations of this study, it was concluded that lay participants assigned higher esthetic ratings than dental professionals for virtual video mock‐ups of themselves and of unknown individuals. Comparisons between self‐evaluation and evaluation of anonymized individuals showed no consistent differences among dentists, while several item‐level differences were observed among lay participants. Compared with natural dentition, ratings for tooth color and tooth alignment increased after viewing the virtual mock‐up, while other items did not change significantly.

## Funding

The authors have nothing to report.

## Conflicts of Interest

The authors declare no conflicts of interest.

## Data Availability

The data that support the findings of this study are available from the corresponding author upon reasonable request.
